# aLFQ: an R-package for estimating absolute protein quantities from label-free LC-MS/MS proteomics data

**DOI:** 10.1093/bioinformatics/btu200

**Published:** 2014-04-20

**Authors:** George Rosenberger, Christina Ludwig, Hannes L. Röst, Ruedi Aebersold, Lars Malmström

**Affiliations:** ^1^Department of Biology, Institute of Molecular Systems Biology, ETH Zurich, Zurich, Switzerland, ^2^PhD Program in Systems Biology, University of Zurich and ETH Zurich, Zurich, Switzerland and ^3^Faculty of Science, University of Zurich, Zurich, Switzerland

## Abstract

**Motivation:** The determination of absolute quantities of proteins in biological samples is necessary for multiple types of scientific inquiry. While relative quantification has been commonly used in proteomics, few proteomic datasets measuring absolute protein quantities have been reported to date. Various technologies have been applied using different types of input data, e.g. ion intensities or spectral counts, as well as different absolute normalization strategies. To date, a user-friendly and transparent software supporting large-scale absolute protein quantification has been lacking.

**Results:** We present a bioinformatics tool, termed aLFQ, which supports the commonly used absolute label-free protein abundance estimation methods (TopN, iBAQ, APEX, NSAF and SCAMPI) for LC-MS/MS proteomics data, together with validation algorithms enabling automated data analysis and error estimation.

**Availability and implementation:** aLFQ is written in R and freely available under the GPLv3 from CRAN (http://www.cran.r-project.org). Instructions and example data are provided in the R-package. The raw data can be obtained from the PeptideAtlas raw data repository (PASS00321).

**Contact:** lars.malmstroem@imsb.biol.ethz.ch

**Supplementary information:** Supplementary data are available at Bioinformatics online.

## 1 INTRODUCTION

A variety of quantitative proteomic methods have been established to measure the relative abundance of proteins across samples. Although relative quantification methods are useful to compare the same proteins between multiple biological samples, they do not provide the possibility to directly compare the data with other datasets or compare different proteins within a dataset with each other and they, by definition, do not provide absolute quantitative data. Further, specific applications, such as differential equation-based modeling of biological systems or determination of subunit stoichiometry of protein complexes depend on absolute protein quantities.

The current gold standard for LC-MS/MS–based absolute protein quantification is the use of stable isotope-labeled standard (SIS) peptides or proteins in precisely determined concentrations (Brun *et al.*, 2009). These standards are spiked into the biological sample of interest and the absolute concentration of the endogenous peptides, and proteins can directly be determined by calculating the ratio of the measured intensities of the spiked-in heavy and the endogenous light forms. For economic reasons, usually only few proteins are quantified using SIS peptides in a single study. To overcome this limitation, multiple absolute label-free methods have been developed in recent years, which allow the estimation of absolute protein abundances for all or a significant fraction of the identified proteins (Lu *et al.*, 2007; Ludwig *et al.*, 2012; Malmström *et al.*, 2009; Schwanhausser *et al.*, 2011; Silva *et al.*, 2006). For a recent discussion and comparison of the methods, see Ahrné *et al.*, (2013). What these methods have in common is that they either use the linear log–log correlation between absolute protein abundance and experimentally estimated protein intensity or an estimate of the total protein concentration of the sample. However, they differ in their protein intensity inference strategy and to date each requires its own computational framework. Here, we provide aLFQ, an open-source implementation of algorithms supporting the estimation of protein quantities by any of the aforementioned methods, and additionally provide automated workflows for data analysis and error estimation.

## 2 IMPLEMENTATION

**Fig. 1.**
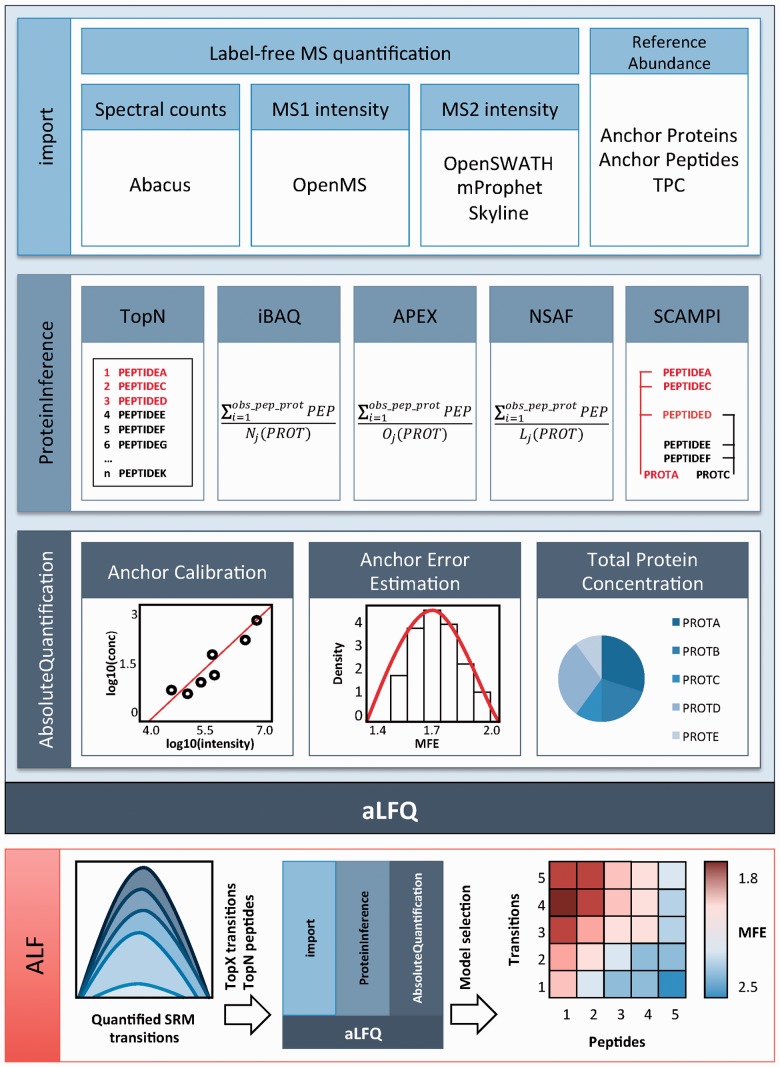
Diagram for exemplary aLFQ workflow with TopX transition and TopN peptide model selection to mediate estimation of protein abundance using SIS peptides. 1. import: generates a generic aLFQ input data structure. 2. ProteinInference: different protein intensity estimation methods can be used to infer protein intensities from measured peptides and transitions. 3. AbsoluteQuantification: using SIS peptides, a model is built and cross-validation is conducted to examine the performance. 4. ALF: different models for varying numbers of transitions and peptides are generated and evaluated and the model with the smallest MFE is selected

aLFQ was implemented in R as a modular S3 package. An example workflow for model selection, depicting the individual functions and sequential arrangement, is shown as diagram in Figure 1. Detailed information on the various workflows and example datasets are provided in the Supplementary Material as well as in the R-package itself.

aLFQ consists of three main modules. The import module provides unified access to the results of common proteomic quantification tools (see Supplementary Material). In addition, an input table with the SIS anchor peptides or anchor proteins and sample-specific absolute abundances or an estimate of the total protein concentration is required.

The ProteinInference module enables inference of protein quantities from precursor intensities, transition intensities or spectral counts. If the dataset contains targeted proteomics data, the paired precursor and fragment ion signals, the transitions are first summarized to the precursor level using one of multiple algorithms. To summarize precursor intensities or spectral counts to protein intensities, the TopN (Ludwig *et al.*, 2012; Malmström *et al.*, 2009; Silva *et al.*, 2006), iBAQ (Schwanhausser *et al.*, 2011), APEX (Lu *et al.*, 2007), NSAF (Zybailov *et al.*, 2006) and SCAMPI (Gerster *et al.*, 2014) methods are provided, enabling direct comparability of the quantitative results.

The AbsoluteQuantification module provides absolute protein-abundance estimation from a linear correlation of a set of predefined anchor proteins or peptides. For this, label-free anchor protein intensities and independently determined accurate anchor protein concentrations are both log transformed and a first order linear least-squares regression is calculated. The abundance of all other proteins in the dataset can be estimated based on this regression. The error of the abundance estimation arises from biological and technical variation as well from the protein and peptide intensity estimators. To estimate the error of the predicted protein concentrations, bootstrapping and Monte Carlo cross-validation are performed, with minimization of the mean-fold error (MFE) as objective function.

## 3 EXAMPLE APPLICATION

An example dataset was produced for this study and is delivered with the aLFQ R-package. The Universal Proteomic Standard 2 (UPS2, Sigma-Aldrich, St. Louis, MO, USA) consists of 48 proteins spanning a dynamic range of five orders of magnitude in bins of eight proteins. The sample was measured in a complex background in shotgun and targeted MS modes (see Supplementary Material). The example data can be accessed using the following commands:

library(aLFQ)

data(UPS2MS)

An exemplary integrated workflow termed ALF (Fig. 1) (Ludwig *et al.*, 2012) conducting peptide and protein inference model selection can be executed with the following command:

ALF(UPS2_SRM)

The workflow evaluates the performance for each combination of TopX transitions and TopN peptides. The resulting MFEs are depicted in a levelplot, and the model with the lowest error is selected for estimation of the concentrations of the target proteins.

## 4 CONCLUSION

aLFQ enables automated absolute label-free protein abundance estimation based on input data from various mass spectrometric measurement modes and analysis software tools. Different quantification methods can be applied in a single framework, and thanks to its implementation in the statistical programming language R, it is accessible to a wide audience of biologists and bioinformaticians. Thus, aLFQ enables easy and fast comparison and selection of the most suitable quantification method and additionally provides an estimation of the absolute abundance estimation error.

*Funding*: G.R. was funded by the Swiss Federal Commission for Technology and Innovation CTI (13539.1 PFFLI-LS), H.L.R. was funded by ETH (ETH-30 11-2), R.A. was funded by PhosphonetX project of SystemsX.ch, the advanced European Research Council grant Proteomics v3.0 (233226) and the Swiss National Science Foundation.

*Conflict of Interest*: none declared.

## Supplementary Material

Supplementary Data
