# Supplementary material for: aLFQ: an R-package for estimating absolute protein quantities from label-free LC-MS/MS proteomics data
Source: Bioinformatics. 2014 Apr 20;30(17):2511–3. doi: 10.1093/bioinformatics/btu200 (PMC4147881; doi:10.1093/bioinformatics/btu200)
Supplement: Supplementary Data [file supp_30_17_2511__index.html]

aLFQ: An R-package for estimating absolute protein quantities from label-free LC-MS/MS proteomics data — aLFQ: an R-package for estimating absolute protein quantities from label-free LC-MS/MS proteomics data — aLFQ: an R-package for estimating absolute protein quantities from label-free LC-MS/MS proteomics data — Supplementary Data 

# aLFQ: an R-package for estimating absolute protein quantities from label-free LC-MS/MS proteomics data

## Supplementary Data

files

**Files in this Data Supplement:**

- Supplementary Data - pdf file
- Supplementary Data - pdf file
