# Supplementary material for: aLFQ: an R-package for estimating absolute protein quantities from label-free LC-MS/MS proteomics data
Source: Bioinformatics. 2014 Apr 20;30(17):2511–3. doi: 10.1093/bioinformatics/btu200 (PMC4147881; doi:10.1093/bioinformatics/btu200)
Supplement: Supplementary Data [file supp_btu200_aLFQ-manual.pdf]

Package ‘aLFQ’

February 20, 2014

Type Package

Title An R-package for estimating absolute protein quantities from label-free LC-MS/MS proteomics data

Version 1.3.1

Date 2014-02-20

Author aLFQ: George Rosenberger, Hannes Roest, Christina Ludwig. AAindex: Barry Grant.

Maintainer George Rosenberger <rosenberger@imsb.biol.ethz.ch>

Depends R (>= 2.15.0)

Imports data.table, plyr, caret, seqinr

Suggests lattice, randomForest, ROCR, reshape2, testthat, protiq

Description
Determination of absolute protein quantities is necessary for multiple applications, such as mechanistic modeling of biological systems. Quantitative liquid chromatography tandem mass spectrometry (LC-MS/MS) proteomics can measure relative protein abundance on a system-wide scale. To estimate absolute quantitative information using these relative abundance measurements requires additional information such as heavy-labeled references of known concentration. Multiple methods have been using different references and strategies; some are easily available whereas others require more effort on the users end. Hence, we believe the field might benefit from making some of these methods available under an automated framework, which also facilitates validation of the chosen strategy. We have implemented the most commonly used absolute label-free protein abundance estimation methods for LC-MS/MS modes quantifying on either MS1-, MS2-levels or spectral counts together with validation algorithms to enable automated data analysis and error estimation. Specifically, we used Monte-carlo cross-validation and bootstrapping for model selection and imputation of proteome-wide absolute protein quantity estimation. Our open-source software is written in the statistical programming language R and validated and demonstrated on a synthetic sample.

License GPL (>= 3)

URL http://www.imsb.ethz.ch

NeedsCompilation no

Repository CRAN

R topics documented:

aLFQ-package 2
aa.index 3
AbsoluteQuantification 4
ALF 6
APEX 8
apexFeatures 10
APEXMS 11
import 12
LUDWIGMS 14
PeptideInference 15
ProteinInference 16
proteotypic 19
UPS2MS 20

Index **21**

---

aLFQ-package *aLFQ*

Description

An R-package for estimating absolute protein quantities from label-free LC-MS/MS proteomics data.

Details

Package:	aLFQ
Type:	Package
Version:	1.3.1
Date:	2014-02-20
Author:	George Rosenberger, Hannes Roest, Christina Ludwig. AAindex: Barry Grant.
Maintainer:	George Rosenberger <rosenberger@imsb.biol.ethz.ch>
Depends:	R (>= 2.15.0)
Imports:	data.table, plyr, caret, seqinr
Suggests:	lattice, randomForest, ROCR, reshape2, testthat, protiq
License:	GPL version 3 or newer
URL:	http://www.imsb.ethz.ch
NeedsCompilation:	no
Repository:	CRAN

Determination of absolute protein quantities is necessary for multiple applications, such as mechanistic modeling of biological systems. Quantitative liquid chromatography tandem mass spectrometry (LC-MS/MS) proteomics can measure relative protein abundance on a system-wide scale. To estimate absolute quantitative information using these relative abundance measurements requires additional information such as heavy-labeled references of known concentration. Multiple methods have been using different references and strategies; some are easily available whereas others require more effort on the users end. Hence, we believe the field might benefit from making some of these methods available under an automated framework, which also facilitates validation of the chosen

strategy. We have implemented the most commonly used absolute label-free protein abundance estimation methods for LC-MS/MS modes quantifying on either MS1-, MS2-levels or spectral counts together with validation algorithms to enable automated data analysis and error estimation. Specifically, we used Monte-carlo cross-validation and bootstrapping for model selection and imputation of proteome-wide absolute protein quantity estimation. Our open-source software is written in the statistical programming language R and validated and demonstrated on a synthetic sample.

See Also

`import`, `ProteinInference`, `AbsoluteQuantification`, `ALF`, `APEX`, `apexFeatures`, `proteotypic`

Examples

```
## Not run: help(package="aLFQ")
```

`aa.index` *AAindex: Amino Acid Index Database*

Description

A collection of published indices, or scales, of numerous physicochemical and biological properties of the 20 standard aminoacids (Release 9.1, August 2006).

Usage

```
data(aa.index)
```

Format

A list of 544 named indeces each with the following components:

1. H character vector: Accession number.
2. D character vector: Data description.
3. R character vector: LITDB entry number.
4. A character vector: Author(s).
5. T character vector: Title of the article.
6. J character vector: Journal reference.
7. C named numeric vector: Correlation coefficients of similar indeces (with coefficients of 0.8/-0.8 or more/less). The correlation coefficient is calculated with zeros filled for missing values.
8. I named numeric vector: Amino acid index data.

Source

'AAIndex' was obtained from:
`ftp://ftp.genome.ad.jp/pub/db/genomenet/aaindex/aaindex1`
For a description of the 'AAindex' database see:
`http://www.genome.jp/aaindex/`.

References

Grant, B.J. et al. (2006) *Bioinformatics* **22**, 2695–2696.

'AAIndex' is the work of Kanehisa and co-workers:
Kawashima and Kanehisa (2000) *Nucleic Acids Res.* **28**, 374;
Tomii and Kanehisa (1996) *Protein Eng.* **9**, 27–36;
Nakai, Kidera and Kanehisa (1988) *Protein Eng.* **2**, 93–100.

Examples

```
## Load AAindex data
data(aa.index)

## Find all indeces described as "volume"
ind <- which(sapply(aa.index, function(x)
                    length(grep("volume", x$D, ignore.case=TRUE)) != 0))

## find all indeces with author "Kyte"
ind <- which(sapply(aa.index, function(x) length(grep("Kyte", x$A)) != 0))

## examine the index
aa.index[[ind]]$I

## find indeces which correlate with it
all.ind <- names(which(Mod(aa.index[[ind]]$C) >= 0.88))

## examine them all
sapply(all.ind, function (x) aa.index[[x]]$I)
```

AbsoluteQuantification *Absolute label-free quantification of mass spectrometry proteomics experiments*

Description

Absolute label-free quantification of mass spectrometry proteomics experiments.

Usage

```
## Default S3 method:
AbsoluteQuantification(data, total_protein_concentration = 1, ...)
## S3 method for class 'AbsoluteQuantification'
cval(object, cval_method = "mc", mcx = 1000, ...)
## S3 method for class 'AbsoluteQuantification'
print(x, ...)
## S3 method for class 'AbsoluteQuantification'
plot(x, ...)
## S3 method for class 'AbsoluteQuantification'
hist(x, ...)
## S3 method for class 'AbsoluteQuantification'
pivot(x, ...)
## S3 method for class 'AbsoluteQuantification'
export(x, file, ...)
```

Arguments

data	a mandatory data frame containing the columns "run_id", "protein_id", "response", and "concentration" as generated by ProteinInference. The id column can be defined in any format, while the "response" and "concentration" columns need to be numeric and in non-log form. The data may contain calibration data (with numeric "concentration" and test data (with "concentration" = "?"))
total_protein_concentration	the total protein concentration in the sample in any unit. This will be used for the normalized protein and concentration columns.
object	an AbsoluteQuantification object.
cval_method	a method for doing crossvalidation: "boot" (bootstrapping), "mc" (monte carlo cross-validation), "loo" (leaving-one-out).
mcx	a positive integer value of the number of folds for cross-validation.
file	the location of the output csv file.
x	an AbsoluteQuantification object.
...	future extensions.

Details

If absolute quantity estimation based on anchor peptides or proteins is demanded, the calibration peptide or protein abundance must be provided. Both estimated calibration protein intensities and separately determined calibration protein concentrations are log transformed and a first order linear least-squares regression of this log-log data is calculated. The abundance of the target proteins is predicted based on this regression. The error of the regression arises from biological and technical variation as well from the protein and peptide intensity estimators. To perform model selection and to estimate the error of the predicted protein concentrations, bootstrapping and Monte Carlo cross-validation as suggested (Malmstrom et al., 2009; Ludwig et al., 2012) were implemented. For both methods, the objective function is the minimization of the mean fold-error.

If, on the other hand, the total protein concentration per cell is supplied in proteome-wide experiments, the absolute protein concentrations are estimated by normalization of the MS intensities or spectral counts to this number (Lu et al., 2006).

Value

An object of class AbsoluteQuantification.

Author(s)

George Rosenberger <rosenberger@imsb.biol.ethz.ch>

References

Malmstrom, J. et al. *Proteome-wide cellular protein concentrations of the human pathogen Leptospira interrogans.* Nature 460, 762-765 (2009).

Ludwig, C., Claassen, M., Schmidt, A. & Aebersold, R. *Estimation of Absolute Protein Quantities of Unlabeled Samples by Selected Reaction Monitoring Mass Spectrometry.* Molecular & Cellular Proteomics 11, M111.013987-M111.013987 (2012).

Lu, P., Vogel, C., Wang, R., Yao, X. & Marcotte, E. M. *Absolute protein expression profiling estimates the relative contributions of transcriptional and translational regulation.* Nat Biotech 25, 117-124 (2006).

See Also

import, ProteinInference, ALF, APEX, apexFeatures, proteotypic

Examples

```
data(UPS2MS)

UPS2_SRM<-head(UPS2_SRM,100) # Remove this line for real applications
data_PI <- ProteinInference(UPS2_SRM)
data_AQ <- predict(cval(AbsoluteQuantification(data_PI),mcx=2))
print(data_AQ)
plot(data_AQ)
hist(data_AQ)
pivot(data_AQ)
```

ALF *Generate ALF report*

Description

Estimation of Absolute Protein Quantities of Unlabeled Samples by Targeted Mass Spectrometry.

Usage

```
## Default S3 method:
ALF(data, report_filename="ALF_report.pdf",
prediction_filename="ALF_prediction.csv", peptide_methods = c("top"),
peptide_topx = c(1,2,3), peptide_strictness = "loose",
peptide_summary = "mean", transition_topx = c(1,2,3),
transition_strictness = "loose", transition_summary = "sum", fasta = NA,
apex_model = NA, combine_precursors = FALSE, combine_peptide_sequences = FALSE,
consensus_proteins = TRUE, consensus_peptides = TRUE, consensus_transitions = TRUE,
scampi_method = "LSE", scampi_iterations = 10, scampi_outliers = FALSE,
scampi_outliers_iterations = 2, scampi_outliers_threshold = 2,
cval_method = "boot", cval_mcx = 1000, ...)
```

Arguments

data
: a mandatory data frame containing the columns "run_id", "protein_id", "peptide_id", "peptide_sequence", "precursor_charge", "peptide_intensity" and "concentration" are required. For quantification on the transition level, the columns "protein_id", "peptide_id", "transition_id", "peptide_sequence", "precursor_charge", "transition_intensity" and "concentration" are required. The id columns can be defined in any format, while the "_intensity" and "concentration" columns need to be numeric and in non-log form. The data may contain calibration data (with numeric "concentration" and test data (with "concentration" = "?"))

report_filename
: the path and filename of the PDF report.

prediction_filename
: the path and filename of the predictions of the optimal model.

peptide_methods
: a vecter containing a combination of "top", "all", "iBAQ", "APEX", "NSAF" or code"SCAMPI" peptide to protein intensity estimation methods.

peptide_topx
: ("top" only:) a positive integer value of the top x peptides to consider for "top" methods.

peptide_strictness
: ("top" only:) whether peptide_topx should only consider proteins with the minimal peptide number ("strict") or all ("loose").

peptide_summary
: ("top" and "all" only:) how to summarize the peptide intensities: "mean", "median", "sum".

transition_topx
: a positive integer value of the top x transitions to consider for transition to peptide intensity estimation methods.

transition_strictness
: whether transition_topx should only consider peptides with the minimal transition number ("strict") or all ("loose").

transition_summary
: how to summarize the transition intensities: "mean", "median", "sum".

fasta
: ("iBAQ", "APEX", "NSAF" and "SCAMPI" only:) the path and filename to an amino acid fasta file containing the proteins of interest.

apex_model
: ("APEX" only:) The "APEX" model to use (see APEX).

combine_precursors
: whether to sum all precursors of the same peptide.

combine_peptide_sequences
: whether to sum all variants (modifications) of the same peptide sequence.

consensus_proteins
: if multiple runs are provided, select identical proteins among all runs.

consensus_peptides
: if multiple runs are provided, select identical peptides among all runs.

consensus_transitions
: if multiple runs are provided, select identical transitions among all runs.

scampi_method
: (SCAMPI only:) Describes which method should be used for the parameter estimation. Available: method="LSE", method="MLE". See details of runScampi or iterateScampi.

scampi_iterations
: (SCAMPI only:) Only used with scampi_method="MLE". See details of runScampi or iterateScampi.

scampi_outliers
: (SCAMPI only:) Whether runScampi (FALSE) or iterateScampi (TRUE) should be used. See details of runScampi or iterateScampi.

scampi_outliers_iterations
: (SCAMPI only:) Number of estimation/outlier-removal iterations to be performed. See details of iterateScampi.

scampi_outliers_threshold
: (SCAMPI only:) Constant to tune the outlier selection process. See details of iterateScampi.

cval_method
: a method for doing crossvalidation: "boot" (bootstrapping), "mc" (monte carlo cross-validation), "loo" (leaving-one-out).

cval_mcx
: a positive integer value of the number of folds for cross-validation.

...
: future extensions.

Details

The ALF module enables model selection for TopN transitions and peptides for protein quantification (Ludwig et al., 2012). The workflow is completely automated and a report and prediction (using the best model) is generated.

Value

The reports specified in the function call.

Author(s)

George Rosenberger <rosenberger@imsb.biol.ethz.ch>

References

Ludwig, C., Claassen, M., Schmidt, A. & Aebersold, R. *Estimation of Absolute Protein Quantities of Unlabeled Samples by Selected Reaction Monitoring Mass Spectrometry.* Molecular & Cellular Proteomics 11, M111.013987-M111.013987 (2012).

See Also

import, ProteinInference, AbsoluteQuantification, APEX, apexFeatures, proteotypic

Examples

```
## Not run: data(UPS2MS)

## Not run: ALF(UPS2_SRM)

## Not run: data(LUDWIGMS)

## Not run: ALF(LUDWIG_SRM)
```

APEX — *Training, testing and validation of APEX peptide observability models*

Description

Calculating absolute and relative protein abundance from mass spectrometry-based protein expression data.

Usage

```
## Default S3 method:
APEX(data, ...)
## S3 method for class 'APEX'
predict(object, newdata=NULL, ...)
## S3 method for class 'APEX'
cval(object, folds=10, ...)
## S3 method for class 'APEX'
print(x, ...)
## S3 method for class 'APEX'
plot(x, ...)
```

Arguments

`data`	an R object of type "`apexFeatures`".
`object`	an `APEX` object.
`newdata`	an R object of type "`apexFeatures`".
`folds`	a positive integer value of the number of folds for cross-validation.
`x`	an `APEX` object.
`...`	future extensions.

Details

The APEX module is a reimplementation of the original algorithm (Lu et al., 2006; Vogel et al., 2008) using the randomForest package. It requires `apexFeatures` input objects and reports the results in an `APEX` object, which can be used by the `ProteinInference` module for protein quantification.

Value

An object of class `APEX`.

Author(s)

George Rosenberger <rosenberger@imsb.biol.ethz.ch>

References

Lu, P., Vogel, C., Wang, R., Yao, X. & Marcotte, E. M. *Absolute protein expression profiling estimates the relative contributions of transcriptional and translational regulation.* Nat Biotech 25, 117-124 (2006).

Vogel, C. & Marcotte, E. M. *Calculating absolute and relative protein abundance from mass spectrometry-based protein expression data.* Nat Protoc 3, 1444-1451 (2008).

See Also

`import`, `ProteinInference`, `AbsoluteQuantification`, `ALF`, `apexFeatures`, `proteotypic`

Examples

```
set.seed(131)

data(APEXMS)

APEX_ORBI<-head(APEX_ORBI,50) # Remove this line for real applications
APEX_ORBI.af <- apexFeatures(APEX_ORBI)
APEX_ORBI.apex <- APEX(data=APEX_ORBI.af)
print(APEX_ORBI.apex)

APEX_ORBI_cval.apex <- cval(APEX_ORBI.apex, folds=2)
plot(APEX_ORBI_cval.apex)
```

apexFeatures *Calculation of physicochemical amino acid properties for APEX*

Description

Calculation of physicochemical amino acid properties for APEX.

Usage

```
## Default S3 method:
apexFeatures(x, ...)
## S3 method for class 'apexFeatures'
print(x, ...)
```

Arguments

`x`	a mandatory data frame containing the variables in the model. The data frame requires the columns `"peptide_sequence"`, `"apex"`. The data may contain training data (with boolean `"apex"` and test data (with `"apex"=NA`))
`...`	future extensions.

Details

The apexFeatures function computes the APEX or PeptideSieve features (Mallick et al., 2006; Vogel et al., 2008) based on AAindex (Kawashima et al., 2008) and returns them in an apexFeatures object for further usage in the `APEX` module.

Value

An object of class `apexFeatures`.

Author(s)

George Rosenberger <rosenberger@imsb.biol.ethz.ch>

References

Kawashima, S. et al. *AAindex: amino acid index database, progress report 2008.* Nucleic Acids Research 36, D202-5 (2008).

Mallick, P. et al. *Computational prediction of proteotypic peptides for quantitative proteomics.* Nat Biotech 25, 125-131 (2006).

Vogel, C. & Marcotte, E. M. *Calculating absolute and relative protein abundance from mass spectrometry-based protein expression data.* Nat Protoc 3, 1444-1451 (2008).

See Also

`import`, `ProteinInference`, `AbsoluteQuantification`, `ALF`, `APEX`, `proteotypic`

Examples

```
data(APEXMS)

# APEX_ORBI
APEX_ORBI<-head(APEX_ORBI,20) # Remove this line for real applications
APEX_ORBI.af <- apexFeatures(APEX_ORBI)
print(APEX_ORBI.af)

# APEX_LCQ
APEX_LCQ<-head(APEX_LCQ,20) # Remove this line for real applications
APEX_LCQ.af <- apexFeatures(APEX_LCQ)
print(APEX_LCQ.af)
```

APEXMS — *Calculating absolute and relative protein abundance from mass spectrometry-based protein expression data.*

Description

This dataset contains training ThermoFinnigan LTQ-OrbiTrap (ORBI) and ThermoFinnigan Surveyor/DecaXP+ iontrap (LCQ) data for training of an `APEX` classifier. The dataset was generated by Christine Vogel and Edward M. Marcotte (see references).

Usage

```
data(APEXMS)
```

Format

A data.frame with the following components:

1. peptide_sequence character vector: Peptide sequence.
2. apex character vector: observed in experiment: 0 = no; 1 = yes.

Source

The dataset was obtained from:
`http://marcottelab.org/APEX_Protocol/`.

References

Vogel, C. & Marcotte, E. M. *Calculating absolute and relative protein abundance from mass spectrometry-based protein expression data.* Nat Protoc 3, 1444-1451 (2008).

See Also

`import`, `ProteinInference`, `AbsoluteQuantification`, `ALF`, `APEX`, `apexFeatures`, `proteotypic`

Examples

```
data(APEXMS)
```

import — *import of mass spectrometry proteomics data analysis software reports.*

Description

import of mass spectrometry proteomics data analysis software reports.

Usage

```
## Default S3 method:
import(ms_filenames, ms_filetype, concentration_filename=NA,
averageruns=FALSE, sumruns=FALSE, mprophet_cutoff=0.01,
openswath_superimpose_identifications=FALSE, openswath_replace_run_id=FALSE,
openswath_filtertop=FALSE, openswath_removedecoys=TRUE,
peptideprophet_cutoff=0.95, abacus_column="ADJNSAF", pepxml2csv_runsplit="~",
...)
```

Arguments

ms_filenames: the paths and filenames of files to import in a character or array class.

ms_filetype: one of "openswath", "mprophet", "openmslfq", "skyline", "abacus" or "pepxml2csv" filetypes. Multiple files of the same type can be supplied in a vector.

concentration_filename: the filename of a csv with concentrations (in any unit). Needs to have the columns "peptide_id" (or "protein_id") and "concentration".

averageruns: whether different MS runs should be averaged.

sumruns: whether different MS runs should be summed.

mprophet_cutoff: (openswath and mprophet only:) the FDR cutoff (m_score) for OpenSWATH and mProphet reports.

openswath_superimpose_identifications: (openswath only:) enables propagation of identification among several runs if feature alignment or requantification was conducted.

openswath_replace_run_id: whether the run_id of the MS data should be replaced by the filename.

openswath_filtertop: (openswath only:) whether only the top peakgroup should be considered.

openswath_removedecoys: (openswath only:) whether decoys should be removed.

peptideprophet_cutoff: (abacus and pepxml2csv only:) the PeptideProphet probability cutoff for Abacus reports.

abacus_column: (abacus only:) target score: one of "NUMSPECSTOT","TOTNSAF","NUMSPECSUNIQ", "UNIQNSAF","NUMSPECSADJ" or "ADJNSAF".

pepxml2csv_runsplit: (pepxml2csv only:) the separator of the run_id and spectrum_id column.

...: future extensions.

Details

The import function provides unified access to the results of various standard proteomic quantification applications like OpenSWATH (Roest et al., 2014), mProphet (Reiter et al., 2011), OpenMS (Sturm et al., 2008; Weisser et al., 2013),Skyline (MacLean et al., 2010) and Abacus (Fermin et al., 2011). This enables generic application of all further steps using the same data structure and enables extension to support other data formats. If multiple runs, i.e. replicates, are supplied, the averaged or summed values can be used to summarize the experimental data. In addition to the input from the analysis software, an input table with the anchor peptides or proteins and sample specific absolute abundance, or an estimate of the total protein concentration in the sample is required. The endpoint of this step is a unified input data structure.

Value

A standard aLFQ import data frame, either on transition, peptide (precursor) or protein level.

Author(s)

George Rosenberger `<rosenberger@imsb.biol.ethz.ch>`

References

Roest H. L. et al. *A tool for the automated, targeted analysis of data-independent acquisition (DIA) MS-data: OpenSWATH.* Nat Biotech, in press.

Reiter, L. et al. *mProphet: automated data processing and statistical validation for large-scale SRM experiments.* Nat Meth 8, 430-435 (2011).

Sturm, M. et al. *OpenMS - An open-source software framework for mass spectrometry.* BMC Bioinformatics 9, 163 (2008).

Weisser, H. et al. *An automated pipeline for high-throughput label-free quantitative proteomics.* J. Proteome Res. 130208071745007 (2013). doi:10.1021/pr300992u

MacLean, B. et al. *Skyline: an open source document editor for creating and analyzing targeted proteomics experiments.* Bioinformatics 26, 966-968 (2010).

Fermin, D., Basrur, V., Yocum, A. K. & Nesvizhskii, A. I. *Abacus: A computational tool for extracting and pre-processing spectral count data for label-free quantitative proteomic analysis.* PROTEOMICS 11, 1340-1345 (2011).

See Also

`ProteinInference`, `AbsoluteQuantification`, `ALF`, `APEX`, `apexFeatures`, `proteotypic`

Examples

```
import(ms_filenames = system.file("extdata","example_openswath.txt",package="aLFQ"),
ms_filetype = "openswath", concentration_filename=NA,
averageruns=FALSE, sumruns=FALSE, mprophet_cutoff=0.01,
openswath_superimpose_identifications=FALSE, openswath_replace_run_id=FALSE,
openswath_filtertop=FALSE, openswath_removedecoys=TRUE)

import(ms_filenames = system.file("extdata","example_mprophet.txt",package="aLFQ"),
ms_filetype = "mprophet",
concentration_filename = system.file("extdata","example_concentration_peptide.csv",
package="aLFQ"), averageruns=FALSE, sumruns=FALSE, mprophet_cutoff=0.01)
```

```
import(ms_filenames = system.file("extdata","example_openmslfq.csv",package="aLFQ"),
ms_filetype = "openmslfq", concentration_filename=NA, averageruns=FALSE, sumruns=FALSE)

import(ms_filenames = system.file("extdata","example_skyline.csv",package="aLFQ"),
ms_filetype = "skyline",
concentration_filename =
system.file("extdata","example_concentration_protein.csv",package="aLFQ"),
averageruns=FALSE, sumruns=FALSE)

import(ms_filenames = system.file("extdata","example_abacus_protein.txt",package="aLFQ"),
ms_filetype = "abacus", concentration_filename =
system.file("extdata","example_concentration_protein.csv",
package="aLFQ"), averageruns=FALSE, sumruns=FALSE,
peptideprophet_cutoff=0.95, abacus_column="ADJNSAF")
```

LUDWIGMS *Estimation of Absolute Protein Quantities of Unlabeled Samples by Selected Reaction Monitoring Mass Spectrometry..*

Description

This dataset contains the Leptospira interrogans MS data from Ludwig C., et al. (2012).

Usage

```
data(LUDWIGMS)
```

Format

The data structure for LUDWIG_SRM represents a data.frame containing the following column header: `"run_id"` (freetext), `"protein_id"` (freetext), `"peptide_id"` (freetext), `"transition_id"` (freetext), `"peptide_sequence"` (unmodified, natural amino acid sequence in 1-letter nomenclature), `"precursor_charge"` (positive integer value), `"transition_intensity"` (positive non-logarithm floating value) and `"concentration"` (calibration: positive non-logarithm floating value, prediction: "?").

References

Ludwig, C., Claassen, M., Schmidt, A. & Aebersold, R. *Estimation of Absolute Protein Quantities of Unlabeled Samples by Selected Reaction Monitoring Mass Spectrometry.* Molecular & Cellular Proteomics 11, M111.013987-M111.013987 (2012).

See Also

`import`, `ProteinInference`, `AbsoluteQuantification`, `ALF`, `APEX`, `apexFeatures`, `proteotypic`

Examples

```
data(LUDWIGMS)
```

PeptideInference *Peptide inference for aLFQ import data frame*

Description

Peptide inference for aLFQ import data frame.

Usage

```
## Default S3 method:
PeptideInference(data, transition_topx = 3,
transition_strictness = "strict",transition_summary = "sum",
consensus_proteins = TRUE, consensus_transitions = TRUE, ...)
```

Arguments

- `data` a mandatory data frame containing the "protein_id", "peptide_id", "transition_id", "peptide_sequence", "precursor_charge", "transition_intensity" and "concentration" are required. The id columns can be defined in any format, while the "_intensity" and "concentration" columns need to be numeric and in non-log form. The data may contain calibration data (with numeric "concentration" and test data (with "concentration" = "?"))
- `transition_topx` a positive integer value of the top x transitions to consider for transition to peptide intensity estimation methods.
- `transition_strictness` whether transition_topx should only consider peptides with the minimal transition number ("strict") or all ("loose").
- `transition_summary` how to summarize the transition intensities: "mean", "median", "sum".
- `consensus_proteins` if multiple runs are provided, select identical proteins among all runs.
- `consensus_transitions` if multiple runs are provided, select identical transitions among all runs.
- `...` future extensions.

Details

The PeptideInference module provides functionality to infer peptide / precursor quantities from the measured precursor or fragment intensities or peptide spectral counts.

Value

A standard aLFQ import data frame on peptide / precursor level.

Author(s)

George Rosenberger <rosenberger@imsb.biol.ethz.ch>

References

Ludwig, C., Claassen, M., Schmidt, A. & Aebersold, R. *Estimation of Absolute Protein Quantities of Unlabeled Samples by Selected Reaction Monitoring Mass Spectrometry.* Molecular & Cellular Proteomics 11, M111.013987-M111.013987 (2012).

See Also

import, AbsoluteQuantification, ALF, APEX, apexFeatures, proteotypic

Examples

```
data(UPS2MS)

data_PI <- PeptideInference(UPS2_SRM)
print(data_PI)
```

ProteinInference *Protein inference for aLFQ import data frame*

Description

Protein inference for aLFQ import data frame.

Usage

```
## Default S3 method:
ProteinInference(data, peptide_method = "top", peptide_topx = 2,
peptide_strictness = "strict",peptide_summary = "mean", transition_topx = 3,
transition_strictness = "strict",transition_summary = "sum", fasta = NA,
apex_model = NA, combine_precursors = FALSE, combine_peptide_sequences = FALSE,
consensus_proteins = TRUE, consensus_peptides = TRUE,
consensus_transitions = TRUE, scampi_method = "LSE",
scampi_iterations = 10, scampi_outliers = FALSE, scampi_outliers_iterations = 2,
scampi_outliers_threshold = 2, ...)
```

Arguments

data	a mandatory data frame containing the columns "run_id", "protein_id", "protein_intensity", and "concentration" for quantification on the protein level. For quantification on the peptide level, the columns "run_id", "protein_id", "peptide_id", "peptide_sequence", "precursor_charge", "peptide_intensity" and "concentration" are required. For quantification on the transition level, the columns "protein_id", "peptide_id", "transition_id", "peptide_sequence", "precursor_charge", "transition_intensity" and "concentration" are required. The id columns can be defined in any format, while the "_intensity" and "concentration" columns need to be numeric and in non-log form. The data may contain calibration data (with numeric "concentration" and test data (with "concentration" = "?"))
peptide_method	one of "top", "all", "iBAQ", "APEX", "NSAF" or "SCAMPI" peptide to protein intensity estimation methods.

peptide_topx
: ("top" only:) a positive integer value of the top x peptides to consider for "top" methods.

peptide_strictness
: ("top" only:) whether peptide_topx should only consider proteins with the minimal peptide number ("strict") or all ("loose").

peptide_summary
: ("top" and "all" only:) how to summarize the peptide intensities: "mean", "median", "sum".

transition_topx
: a positive integer value of the top x transitions to consider for transition to peptide intensity estimation methods.

transition_strictness
: whether transition_topx should only consider peptides with the minimal transition number ("strict") or all ("loose").

transition_summary
: how to summarize the transition intensities: "mean", "median", "sum".

fasta
: ("iBAQ", "APEX", "NSAF" and "SCAMPI" only:) the path and filename to an amino acid fasta file containing the proteins of interest.

apex_model
: ("APEX" only:) The "APEX" model to use (see APEX).

combine_precursors
: whether to sum all precursors of the same peptide.

combine_peptide_sequences
: whether to sum all variants (modifications) of the same peptide sequence.

consensus_proteins
: if multiple runs are provided, select identical proteins among all runs.

consensus_peptides
: if multiple runs are provided, select identical peptides among all runs.

consensus_transitions
: if multiple runs are provided, select identical transitions among all runs.

scampi_method
: (SCAMPI only:) Describes which method should be used for the parameter estimation. Available: method="LSE", method="MLE". See details of runScampi or iterateScampi.

scampi_iterations
: (SCAMPI only:) Only used with scampi_method="MLE". See details of runScampi or iterateScampi.

scampi_outliers
: (SCAMPI only:) Whether runScampi (FALSE) or iterateScampi (TRUE) should be used. See details of runScampi or iterateScampi.

scampi_outliers_iterations
: (SCAMPI only:) Number of estimation/outlier-removal iterations to be performed. See details of iterateScampi.

scampi_outliers_threshold
: (SCAMPI only:) Constant to tune the outlier selection process. See details of iterateScampi.

...
: future extensions.

Details

The ProteinInference module provides functionality to infer protein quantities from the measured precursor or fragment intensities or peptide spectral counts. If the dataset contains targeted MS2-level data, the paired precursor and fragment ion signals, the transitions, are first summarized to the precursor level. Different methods for aggregation can be specified, e.g. sum, mean or median and a limit for the selection of the most intense transitions can be provided. It is further possible to exclude precursors, which do not have sufficient transitions to fulfill this boundary. To summarize precursor intensities or spectral counts to theoretical protein intensities, the mean, TopN (Silva et al., 2006; Malmstrom et al., 2009; Schmidt et al., 2011; Ludwig et al., 2012), APEX (Lu et al., 2006), iBAQ (Schwanhausser et al., 2011), NSAF (Zybailov et al., 2006) and SCAMPI (Gerster et al., 2014) protein intensity estimators are provided. For APEX, iBAQ, NSAF and SCAMPI, the protein database in FASTA format needs to be supplied. In terms of targeted data acquisition, for both APEX and iBAQ methods all peptides of a protein must be targeted. The results are reported in the same unified data structure as from the previous step

Value

A standard aLFQ import data frame on protein level.

Author(s)

George Rosenberger <rosenberger@imsb.biol.ethz.ch>

References

Silva, J. C., Gorenstein, M. V., Li, G.-Z., Vissers, Johannes P. C. & Geromanos, S. J. *Absolute quantification of proteins by LCMSE: a virtue of parallel MS acquisition.* Mol. Cell Proteomics 5, 144-156 (2006).

Malmstrom, J. et al. *Proteome-wide cellular protein concentrations of the human pathogen Leptospira interrogans.* Nature 460, 762-765 (2009).

Schmidt, A. et al. *Absolute quantification of microbial proteomes at different states by directed mass spectrometry.* Molecular Systems Biology 7, 1-16 (2011).

Ludwig, C., Claassen, M., Schmidt, A. & Aebersold, R. *Estimation of Absolute Protein Quantities of Unlabeled Samples by Selected Reaction Monitoring Mass Spectrometry.* Molecular & Cellular Proteomics 11, M111.013987-M111.013987 (2012).

Lu, P., Vogel, C., Wang, R., Yao, X. & Marcotte, E. M. *Absolute protein expression profiling estimates the relative contributions of transcriptional and translational regulation.* Nat Biotech 25, 117-124 (2006).

Schwanhausser, B. et al. *Global quantification of mammalian gene expression control.* Nature 473, 337-342 (2011).

Zybailov, B. et al. *Statistical Analysis of Membrane Proteome Expression Changes in Saccharomyces c erevisiae.* J. Proteome Res. 5, 2339-2347 (2006).

Gerster S., Kwon T., Ludwig C., Matondo M., Vogel C., Marcotte E. M., Aebersold R., Buhlmann P. *Statistical approach to protein quantification.* Molecular & Cellular Proteomics 13, M112.02445 (2014).

See Also

`import`, `AbsoluteQuantification`, `ALF`, `APEX`, `apexFeatures`, `proteotypic`, `runScampi`, `iterateScampi`

Examples

```
data(UPS2MS)

data_ProteinInference <- ProteinInference(UPS2_SRM)
print(data_ProteinInference)
```

`proteotypic` *Prediction of the flyability of proteotypic peptides*

Description

Prediction of the flyability of proteotypic peptides.

Usage

```
## Default S3 method:
proteotypic(fasta, apex_model, min_aa=4 , max_aa=20, ...)
```

Arguments

`fasta`	a amino acid FASTA file.
`apex_model`	an `APEX` object.
`min_aa`	the minimum number of amino acids for proteotypic peptides.
`max_aa`	the maximum number of amino acids for proteotypic peptides.
`...`	future extensions.

Details

This function provides prediction of the "flyability" of proteotypic peptides using the APEX method (Lu et al., 2006; Vogel et al., 2008). The APEX scores are probabilities that indicate detectability of the peptide amino acid sequence in LC-MS/MS experiments.

Value

A data.frame containing peptide sequences and associated APEX scores.

Author(s)

George Rosenberger `<rosenberger@imsb.biol.ethz.ch>`

References

Lu, P., Vogel, C., Wang, R., Yao, X. & Marcotte, E. M. *Absolute protein expression profiling estimates the relative contributions of transcriptional and translational regulation.* Nat Biotech 25, 117-124 (2006).

Vogel, C. & Marcotte, E. M. *Calculating absolute and relative protein abundance from mass spectrometry-based protein expression data.* Nat Protoc 3, 1444-1451 (2008).

See Also

`import`, `ProteinInference`, `AbsoluteQuantification`, `ALF`, `APEX`, `apexFeatures`

Examples

```
set.seed(131)

data(APEXMS)

APEX_ORBI<-head(APEX_ORBI,20) # Remove this line for real applications
APEX_ORBI.af <- apexFeatures(APEX_ORBI)
APEX_ORBI.apex <- APEX(data=APEX_ORBI.af)

peptides <- proteotypic(fasta=system.file("extdata","example.fasta",package="aLFQ"),
apex_model=APEX_ORBI.apex, min_aa=4 , max_aa=20)
## Not run: print(peptides)
```

UPS2MS *Calculating absolute and relative protein abundance from mass spectrometry-based protein expression data.*

Description

We assessed the performance of aLFQ and the different quantification estimation methods it supports by investigating a commercially available synthetic sample. The Universal Proteomic Standard 2 (UPS2) consists of 48 proteins spanning a dynamic range of five orders of magnitude in bins of eight proteins. The sample was measured in a complex background consisting of Mycobacterium bovis BCG total cell lysate in shotgun and targeted MS modes. Three datasets are available: UPS2_SC (spectral counts), UPS2_LFQ (MS1 intensity), UPS2_SRM (MS2 intensity).

Usage

```
data(UPS2MS)
```

Format

The data structure for UPS2_SRM represents a data.frame containing the following column header: `"run_id"` (freetext), `"protein_id"` (freetext), `"peptide_id"` (freetext), `"transition_id"` (freetext), `"peptide_sequence"` (unmodified, natural amino acid sequence in 1-letter nomenclature), `"precursor_charge"` (positive integer value), `"transition_intensity"` (positive non-logarithm floating value) and `"concentration"` (calibration: positive non-logarithm floating value, prediction: "?").

The data structure for UPS2_LFQ (MS1-level intensity) / UPS2_SC (spectral counts) represents a data.frame containing the columns `"run_id"` (freetext), `"protein_id"` (freetext), `"peptide_id"` (freetext), `"peptide_sequence"` (unmodified, natural amino acid sequence in 1-letter nomenclature), `"precursor_charge"` (positive integer value), `"peptide_intensity"` (positive non-logarithm floating value) and `"concentration"` (calibration: positive non-logarithm floating value, prediction: "?"). It should be noted, that the spectral count value is also represented by `"peptide_intensity"`.

See Also

import, ProteinInference, AbsoluteQuantification, ALF, APEX, apexFeatures, proteotypic

Examples

```
data(UPS2MS)
```

Index

∗Topic **AAIndex**
apexFeatures, 10
∗Topic **ALF**
ALF, 6
∗Topic **APEX**
APEX, 8
apexFeatures, 10
APEXMS, 11
proteotypic, 19
∗Topic **AQUA**
ALF, 6
∗Topic **Abacus**
import, 12
∗Topic **AbsoluteQuantification**
AbsoluteQuantification, 4
∗Topic **LFQ**
import, 12
∗Topic **OpenMS**
import, 12
∗Topic **OpenSWATH**
import, 12
∗Topic **PeptideInference**
PeptideInference, 15
∗Topic **PeptideSieve**
apexFeatures, 10
∗Topic **ProteinInference**
ProteinInference, 16
∗Topic **SCAMPI**
ProteinInference, 16
∗Topic **SIS**
ALF, 6
∗Topic **SRM**
ALF, 6
∗Topic **SWATH**
ALF, 6
∗Topic **Skyline**
import, 12
∗Topic **UPS2**
LUDWIGMS, 14
UPS2MS, 20
∗Topic **aLFQ**
aLFQ-package, 2
∗Topic **absolute**
AbsoluteQuantification, 4
∗Topic **datasets**
aa.index, 3
APEXMS, 11
LUDWIGMS, 14
UPS2MS, 20
∗Topic **flyability**
proteotypic, 19
∗Topic **high-flyers**
proteotypic, 19
∗Topic **label-free**
AbsoluteQuantification, 4
PeptideInference, 15
ProteinInference, 16
∗Topic **mProphet**
import, 12
∗Topic **peptide inference**
PeptideInference, 15
ProteinInference, 16
∗Topic **protein inference**
ProteinInference, 16
∗Topic **proteotypic peptides**
proteotypic, 19
∗Topic **quantification**
AbsoluteQuantification, 4
PeptideInference, 15
ProteinInference, 16

aa.index, 3
AbsoluteQuantification, *3*, 4, *8–11*, *13, 14*, *16*, *18–20*
ALF, *3*, *6*, 6, *9–11*, *13, 14*, *16*, *18–20*
aLFQ *(*aLFQ-package*)*, 2
aLFQ-package, 2
APEX, *3*, *6*, *8*, 8, *10, 11*, *13, 14*, *16*, *18–20*
APEX_LCQ *(*APEXMS*)*, 11
APEX_ORBI *(*APEXMS*)*, 11
apexFeatures, *3*, *6*, *8, 9*, 10, *11*, *13, 14*, *16*, *18–20*
APEXMS, 11

cval *(*AbsoluteQuantification*)*, 4
cval.APEX *(*APEX*)*, 8

export (AbsoluteQuantification), 4

hist.AbsoluteQuantification
(AbsoluteQuantification), 4

import, *3*, *6*, *8–11*, 12, *14*, *16*, *18–20*
iterateScampi, *18*

LUDWIG_SRM (LUDWIGMS), 14
LUDWIGMS, 14

PeptideInference, 15
pivot (AbsoluteQuantification), 4
plot.AbsoluteQuantification
(AbsoluteQuantification), 4
plot.APEX (APEX), 8
predict.AbsoluteQuantification
(AbsoluteQuantification), 4
predict.APEX (APEX), 8
print.AbsoluteQuantification
(AbsoluteQuantification), 4
print.APEX (APEX), 8
print.apexFeatures (apexFeatures), 10
ProteinInference, *3*, *6*, *8–11*, *13*, *14*, 16, *19*, *20*
proteotypic, *3*, *6*, *8–11*, *13*, *14*, *16*, *18*, 19, *20*

runScampi, *18*

UPS2 (UPS2MS), 20
UPS2_LFQ (UPS2MS), 20
UPS2_SC (UPS2MS), 20
UPS2_SRM (UPS2MS), 20
UPS2MS, 20
