# Supplementary material for: aLFQ: an R-package for estimating absolute protein quantities from label-free LC-MS/MS proteomics data
Source: Bioinformatics. 2014 Apr 20;30(17):2511–3. doi: 10.1093/bioinformatics/btu200 (PMC4147881; doi:10.1093/bioinformatics/btu200)
Supplement: Supplementary Data [file supp_btu200_aLFQ_SI.pdf]

aLFQ: An R-package for estimating absolute protein quantities from label-free LC-MS/MS proteomics data

George Rosenberger [1,2], Christina Ludwig [1], Hannes L. Röst [1,2], Ruedi Aebersold[1,3] and Lars Malmström[1*]

[1]Department of Biology, Institute of Molecular Systems Biology, ETH Zurich, CH-8093 Zurich, Switzerland, [2]Ph.D. Program in Systems Biology, University of Zurich and ETH Zurich, CH-8057 Zurich, Switzerland, [3]Faculty of Science, University of Zurich, CH-8057 Zurich, Switzerland

[*]to whom correspondence should be addressed

1 Experimental measurements

We assessed the performance of aLFQ and the different quantification estimation methods it supports by investigating a commercially available synthetic sample. The Universal Proteomic Standard 2 (UPS2) consists of 48 proteins spanning a dynamic range of five orders of magnitude in bins of eight proteins. The sample was measured in a complex background consisting of *Mycobacterium bovis* BCG total cell lysate in shotgun and targeted MS modes. The processed datasets are available in the aLFQ R-package and can be accessed by the command:

```
library(aLFQ)
?UPS2MS
```

1.1 UPS1 and UPS2 sample preparation

The Universal Proteomics Standard (UPS, Sigma-Aldrich, St. Louis, MO, USA) is a set of 48 equimolar human proteins, with a total of 592 theoretical tryptic peptides with at least 8 amino acids. These proteins were quantified by amino acid analysis and are unlabeled. The UPS1 sample consists of the proteins in equimolar concentration, whereas in the UPS2 sample the same 48 human proteins are diluted in bins of 8 proteins of equal concentrations to span 5 orders of magnitude.

The samples UPS1 and UPS2 were both purchased from Sigma-Aldrich in lyophilized form in quantities of 5 pmol per protein for UPS1 (~6.4 µg total protein) and 10.6 µg total protein for UPS2 (50 pmol to 500 amol). In a first step both samples were resuspended in 40 µL of denaturation buffer (8 M urea, 100 mM NH4HCO3, pH 8.0). In the case of UPS1 1.1 µg of total protein (860 fmol per protein) were mixed with 4.4 µg of a *Mycobacterium bovis* BCG total cell lysate, while for UPS2 1.8 µg of UPS proteins were mixed with 7.2 µg of cellular lysate. Subsequently all proteins were reduced with 5 mM tris(2-carboxyethyl)phosphine (TCEP), alkylated with 40 mM of iodacetamide, 5-times diluted with 100 mM NH_4HCO_3 (to 1.6 M urea) and digested at 30°C for 16 hours with sequence grade modified trypsin (protein to enzyme ratio 50:1). Trypsin activity was quenched by adding trifluoroacetic acid (TFA) to adjust pH to < 2 and peptides were purified using C18 Micro-Spin columns with loading capacity

5 to 50 µg (The Nest Group Inc., Southborough, MA, USA). After elution with 40% acetonitrile (ACN), 60% H_2O and 0.1% TFA the samples were dried and resuspended in H_2O and 0.1% formic acid (FA), resulting in UPS protein concentrations of 78.4 fmol/µL for UPS1 (0.4 µg/µl BCG cell lysate) and 490 fmol/µl down to 4.9 amol/µl for UPS2 (0.4 µg/µl BCG cell lysate).

1.2 Shotgun mass spectrometry

The samples UPS1 and UPS2 were measured on a hybrid LTQ-Orbitrap mass spectrometer (Thermo Fisher, San Jose, CA, USA), equipped with a nano-electrospray ion source and a NanoLC-2Dplus HPLC system (Eksigent, Dublin, CA, USA). The system was coupled with a 10 cm and 75 µm diameter column, which was packed with a Magic C18 AQ 3 µm resin (Michrom Bio-Resources, Auburn, CA, USA). For the UPS1 sample each UPS protein was injected at a concentration of 78.6 fmol on column, while in the UPS2 sample a concentration range from 900 fmol to 9 atmol on column was applied. A linear 60 min (UPS1) or 120 min (UPS2) gradient of 5-35% buffer B (98% ACN, 2% H_2O, 0.1% formic acid) was used to separate the peptides at a flow rate of 300 nL/min. For MS/MS data acquisition, 5 data-dependent MS/MS scans were acquired in the linear ion trap for each MS1 scan. The latter was acquired at 60,000 full width at half maximum (FWHM) nominal resolution settings. A minimum signal threshold was defined at 250 counts (UPS1) or 150 counts (UPS2). The applied mass scan range was 350.00 to 1600.00 m/z. The dynamic exclusion function was enabled with an exclusion duration of 30 s and an exclusion list size of 500 (UPS1) or 300 (UPS2). Only peptides with an assigned charge state of 2+ or higher were enabled for fragmentation, while unassigned or singly charged states were rejected. All measurements were carried out in technical triplicates.

UPS1	UPS2
chludwig_M1107_273	chludwig_M1202_188
chludwig_M1107_281	chludwig_M1202_189
chludwig_M1107_286	chludwig_M1202_190

Table 1: UPS 1 and UPS2 shotgun measurement file names

The data is available from the PeptideAtlas raw data repository server: http://www.peptideatlas.org/PASS/PASS00321

1.3 Targeted mass spectrometry

Only the UPS2 sample was analyzed by SRM on a TSQ Vantage Triple Quadrupole mass spectrometer (Thermo Fisher, San Jose, CA, USA), equipped with a nano-electrospray ion source and a NanoLC-2Dplus HPLC system (Eksigent, Dublin, CA, USA). The spray voltage was set to 1.35 keV and the heated ion transfer tube was kept at 280°C. The system was coupled with a 10 cm and 75 µm diameter column packed with a Magic C18 AQ 5 µm resin (Michrom Bio-Resources, Auburn, CA, USA). A 40 min linear gradient of 5-46% buffer B (98% ACN, 2% H_2O, 0.1% formic acid) was used to separate the peptides at a flow rate of 300 nL/min. Q1 and Q3 were obtained at 0.7 amu resolution. Argon was used as collision gas at a nominal pressure of 1.5 mTorr. Doubly and triply charged precursor ions were measured and the collision energy was calculated using the following equations:

2+ precursor: CE = 0.034 ∗ (m/z) - 0.848.
3+ precursor: CE = 0.022 ∗ (m/z) + 5.953.

The 48 UPS2 proteins were measured over four injections per sample and the UPS2 sample was acquired in technical triplicates. Assays were generated using a consensus spectral library from the UPS1 shotgun measurements. For each measurement UPS2 proteins spanning a concentration range from 490 fmol down to 4.9 amol were injected on column.

**UPS2**		
chludwig_H110822_416	chludwig_H110822_417	chludwig_H110822_419
chludwig_H110822_420	chludwig_H110822_422	chludwig_H110822_423
chludwig_H110822_425	chludwig_H110822_426	chludwig_H110822_428
chludwig_H110822_429	chludwig_H110822_431	chludwig_H110822_434

Table 2: UPS2 targeted measurement file names

The data is available from the PeptideAtlas raw data repository server: http://www.peptideatlas.org/PASS/PASS00321

1.4 Shotgun data analysis

The spectra were searched with the search engines X!Tandem using the k-score plugin (2011.12.01.1) (Keller *et al.*, 2005), OMSSA (2.1.9) (Geer *et al.*, 2004) and MyriMatch (2.1.138) (Tabb *et al.*, 2007) against the provided database (UPS, Sigma-Aldrich, St. Louis, MO, USA) concatenated with an *M. tuberculosis* database (TubercuList Release 23) (Lew *et al.*, 2011) using Trypsin digestion and allowing 0 missed cleavage. Included was 'Carbamidomethyl (C)' as static modification. The mass tolerances were set to 15 ppm for precursor-ions and 0.4 Da for fragment-ions. The identified peptides were processed and analyzed through the Trans-Proteomic Pipeline (4.6.0) (Deutsch *et al.*, 2010) using PeptideProphet (Keller *et al.*, 2002), iProphet (Shteynberg *et al.*, 2011) and ProteinProphet (Nesvizhskii *et al.*, 2003) scoring. Peptide identifications were reported at FDR of 0.01, corresponding to an iProphet probability of >= 0.85. Label-free quantification using spectral counts was conducted using an in-house developed script: All PSM above an iProphet probability >= 0.85 were selected, corresponding to a peptide FDR of <= 1% and a protein FDR of <= 1%. The label-free quantification pipeline of OpenMS (1.10) was used as described previously (Weisser *et al.*, 2013) using peptide identifications with peptide FDR of <= 1%. Both results were filtered to only contain UPS proteins and peptides and were imported using the aLFQ import functionality with averaging of runs enabled. One outlier peptide with sequence "IECVSAETTEDCIAK" was removed from both datasets manually.

1.5 Targeted data analysis

The raw data from targeted MS experiments was manually analyzed using Skyline (MacLean *et al.*, 2010). A consensus spectral library was generated from the Shotgun data analysis results of UPS1 using SpectraST (4.0) (Lam *et al.*, 2008) and used for transition selection in Skyline. In total 137 peptides and 928 transitions were annotated as true positive. The data is available from the

Panorama Skyline server:
https://daily.panoramaweb.org/labkey/project/Aebersold/ludwig/aLFQ/begin.view?

2 Example application

2.1 Installation of aLFQ

Please note that aLFQ requires R version 2.15.0 or greater. The SCAMPI protein inference method further requires the installation of two Bioconductor packages. The packages can be installed by the following commands in R:

```
source("http://bioconductor.org/biocLite.R")
biocLite("RBGL")
biocLite("graph")
```

To install aLFQ, execute the following command in R afterwards:

```
install.packages(“aLFQ”, dependencies=TRUE)
```

2.2 Model selection for UPS2 SRM dataset

To conduct a full model selection on the example UPS2 SRM dataset, execute the following steps in an R session with installed aLFQ:

```
library(aLFQ)

## 1. Step: Training of an APEX model
# Loads APEX data into session. See ?APEXMS for
information on the dataset.
data(APEXMS)

# Generates physicochemical features for APEX_ORBI
dataset. See ?apexFeatures for information on the method.
APEX_ORBI.af <- apexFeatures(APEX_ORBI)

# Trains APEX model. See ?APEX for information on the
method.
APEX_ORBI.apex <- APEX(data=APEX_ORBI.af)

## 2. Step: Loading of example data data.
# Loads UPS2 data into session. See ?UPS2MS for
information on the dataset.
data(UPS2MS)

# For other datasets, the import method can be used.
Please refer to ?import for parameters for other
quantfication tools.
# import(ms_filenames =
system.file("extdata","example_skyline.csv",package="aLFQ
"), ms_filetype = "skyline", concentration_filename =
system.file("extdata","example_concentration_protein.csv"
,package="aLFQ"), averageruns=FALSE, sumruns=FALSE)

## 3. Step: Model selection and absolute abundance
estimation
```

```
# Conducts model selection as described in the main text
on the UPS2 SRM data but with additional protein
inference methods. See ?ALF for further information on
the parameters.
ALF(UPS2_SRM, report_filename="ALF_SRM_report.pdf",
prediction_filename="ALF_SRM_prediction.csv",
peptide_methods = c("top", "all", "iBAQ", "APEX", "NSAF",
"SCAMPI"), peptide_topx = c(1,2,3), peptide_strictness =
"loose", peptide_summary = "mean", transition_topx =
c(1,2,3), transition_strictness = "strict",
transition_summary = "sum", fasta =
system.file("extdata","UPS2.fasta",package="aLFQ"),
apex_model = APEX_ORBI.apex, combine_precursors = TRUE,
combine_peptide_sequences = TRUE)

# Conducts the same model selection on the UPS2 spectral
count data.
ALF(UPS2_SC, report_filename="ALF_SC_report.pdf",
prediction_filename="ALF_SC_prediction.csv",
peptide_methods = c("top", "all", "iBAQ", "APEX", "NSAF",
"SCAMPI"), peptide_topx = c(1,2,3), peptide_strictness =
"loose", peptide_summary = "mean", transition_topx =
c(1), transition_strictness = "strict",
transition_summary = "sum", fasta =
system.file("extdata","UPS2.fasta",package="aLFQ"),
apex_model = APEX_ORBI.apex, combine_precursors = TRUE,
combine_peptide_sequences = TRUE)

# Conducts the same model selection on the UPS2 MS1
intensity LFQ data.
ALF(UPS2_LFQ, report_filename="ALF_LFQ_report.pdf",
prediction_filename="ALF_LFQ_prediction.csv",
peptide_methods = c("top", "all", "iBAQ", "APEX", "NSAF",
"SCAMPI"), peptide_topx = c(1,2,3), peptide_strictness =
"loose", peptide_summary = "mean", transition_topx =
c(1), transition_strictness = "strict",
transition_summary = "sum", fasta =
system.file("extdata","UPS2.fasta",package="aLFQ"),
apex_model = APEX_ORBI.apex, combine_precursors = TRUE,
combine_peptide_sequences = TRUE)
```

The output of these three workflows are for each one PDF file with the report of the model selection and one CSV file containing the absolute protein abundance estimates of the protein inference model with the smallest mean fold error.

2.3 Expected Results

Comparing the result reports for the three datasets indicates that different models should be used for different label-free quantification methods. Particularly, the application of iBAQ, NSAF and APEX for SRM datasets is not justified, as not all detectable peptides per protein have been measured. For the UPS2 SRM dataset, the peptide inference method summarizing the three most intense transitions per peptide and the three most intense peptides per proteins

results in the smallest mean fold error, whereas NSAF achieves the best results for spectral counts and iBAQ for MS1 intensities respectively (Fig. 1 – 9).

2.3.1 Results for UPS2 SRM dataset

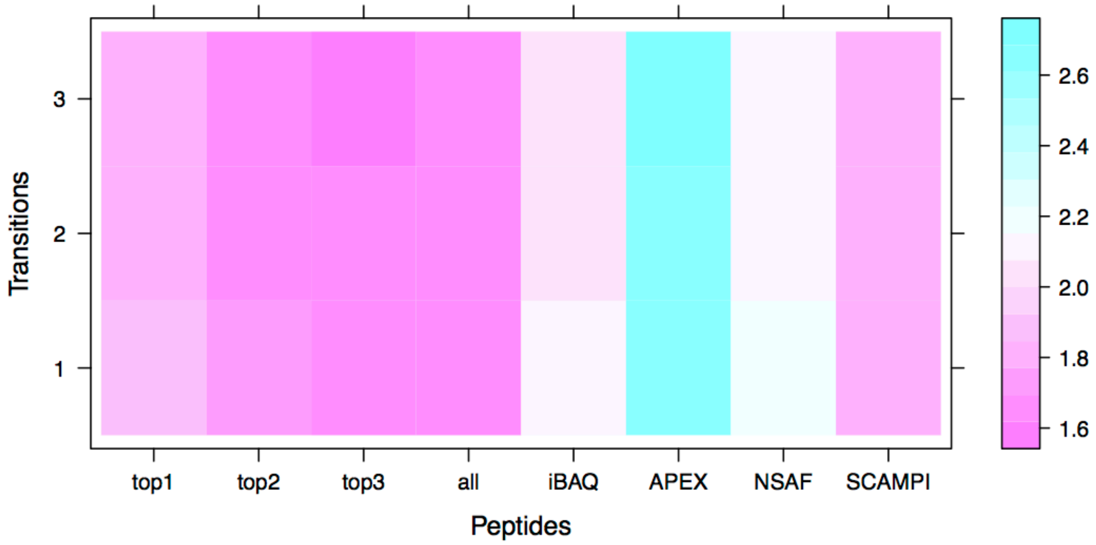

Figure 1: Model selection report for the UPS2_SRM dataset. The TopN variant with three peptides and three proteins performed best. Please note that the SRM dataset with only selected peptides measured does not fulfill the assumptions of iBAQ, APEX & NSAF.

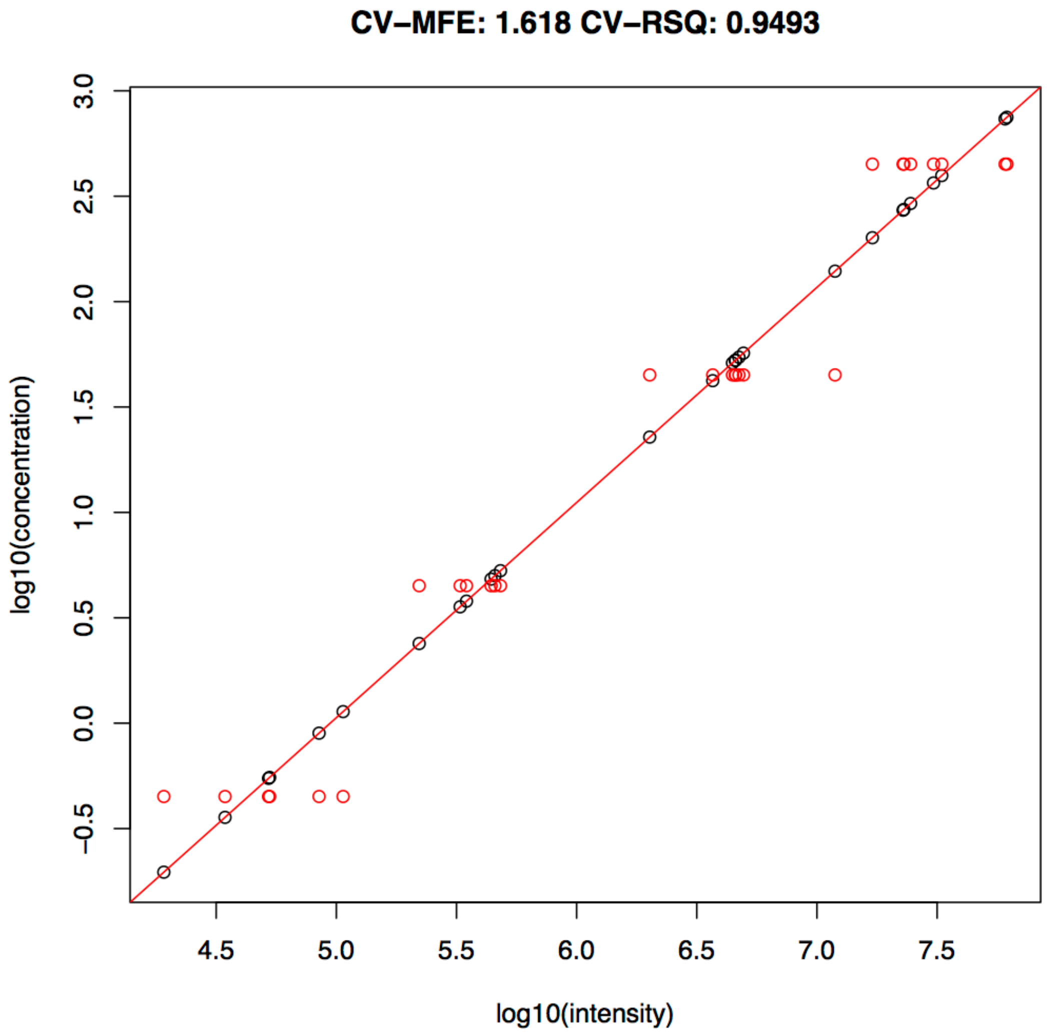

Figure 2: Linear regression plot of log10(intensity) vs log10(concentration) for the UPS2_SRM dataset. The measured proteins span 3 orders of magnitude with a cross-validated mean-fold error of 1.618.

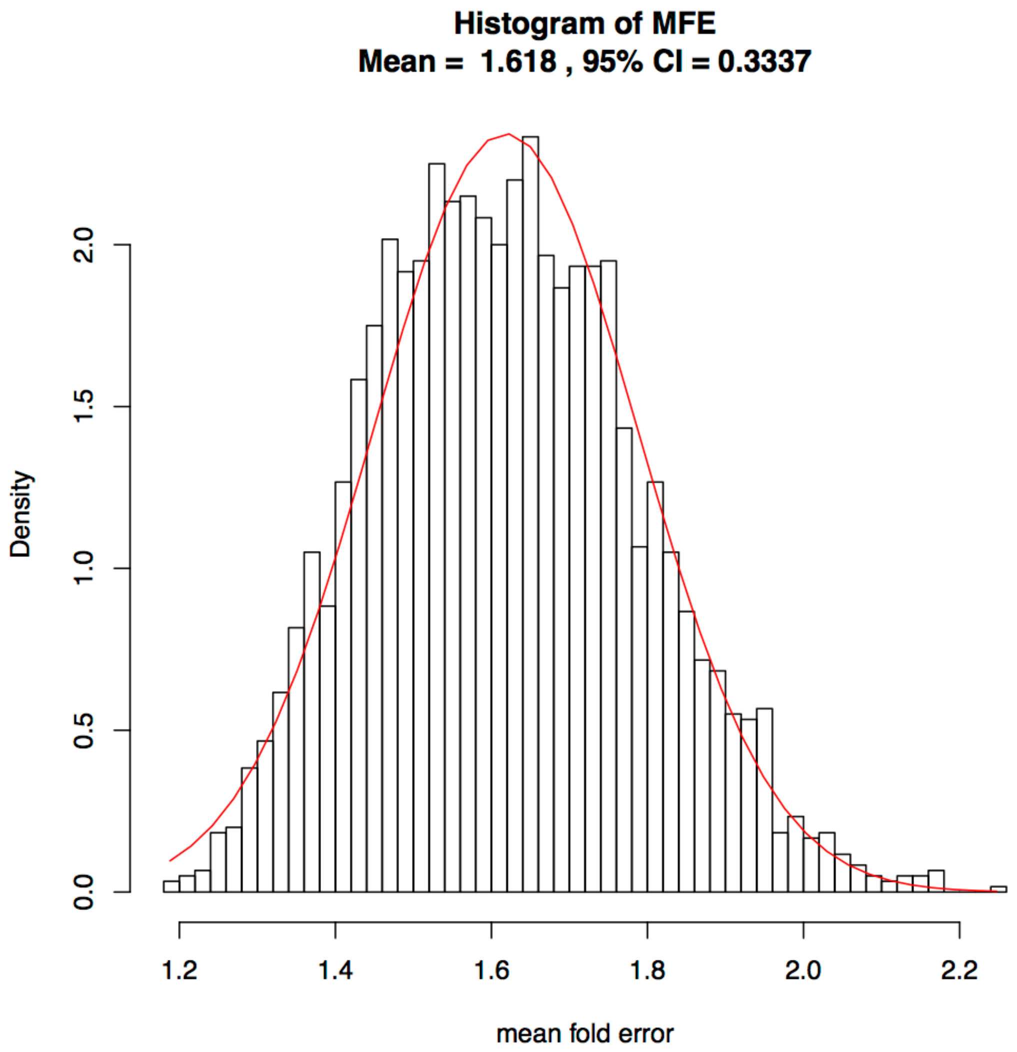

Figure 3: Histogram of the mean fold error for the UPS2_SRM dataset. The 95% confidence interval is 0.3337 with a mean of 1.618.

2.3.2 Results for UPS2 spectral counts dataset

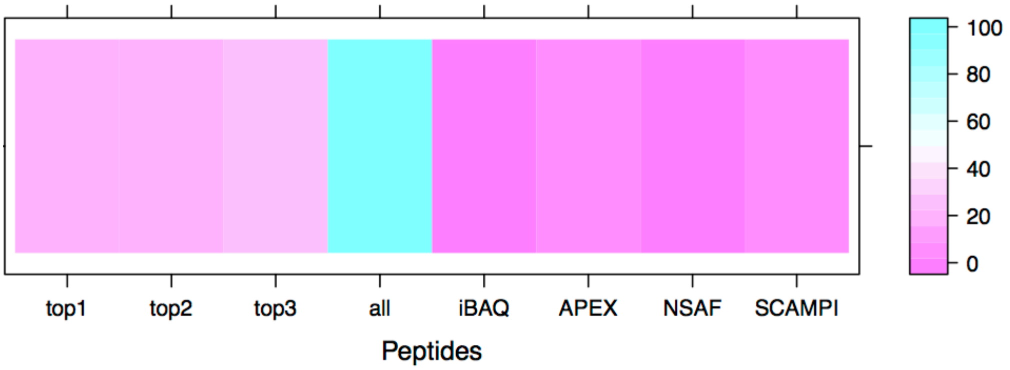

Figure 4: Model selection report for the UPS2_SC dataset. The NSAF protein inference method performed best.

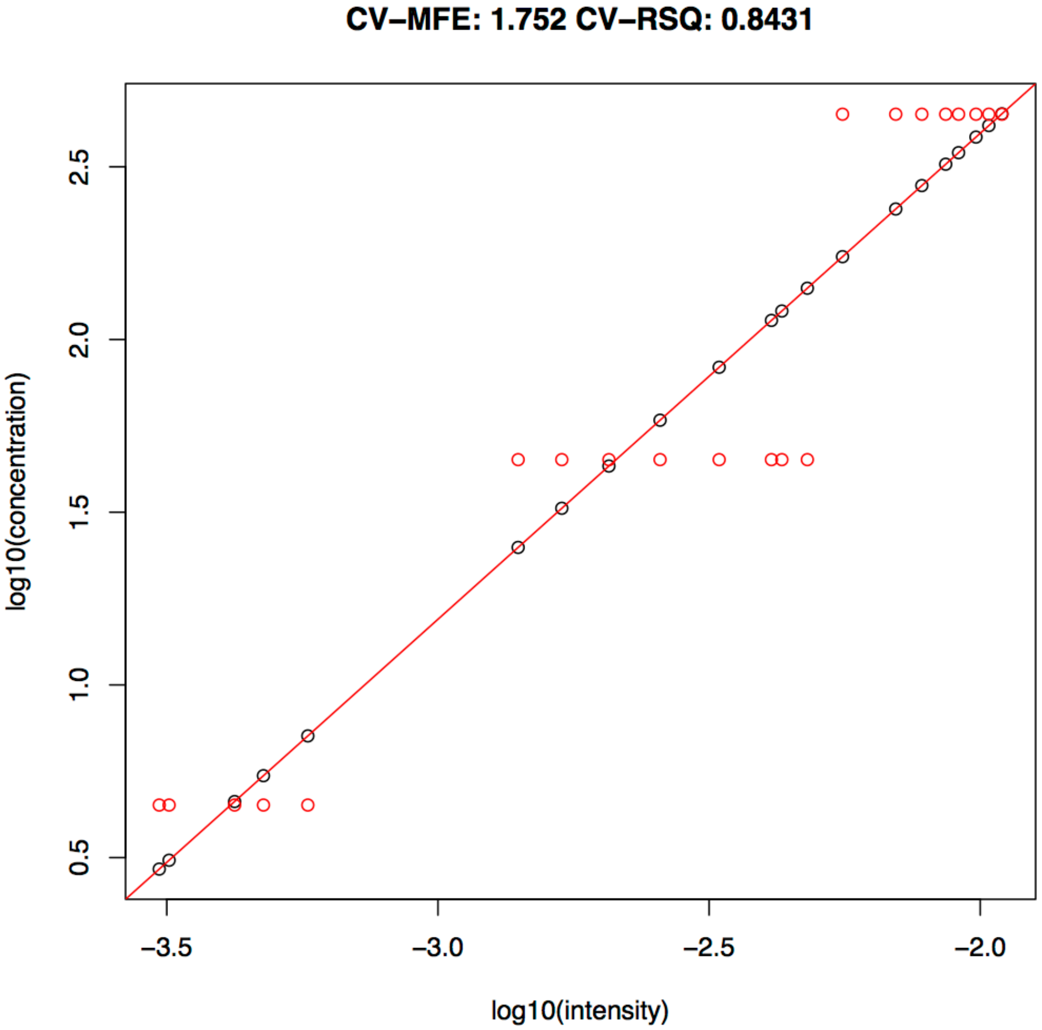

Figure 5: Linear regression plot of log10(intensity) vs log10(concentration) for the UPS2_SC dataset. The measured proteins span 2 orders of magnitude with a cross-validated mean-fold error of 1.752.

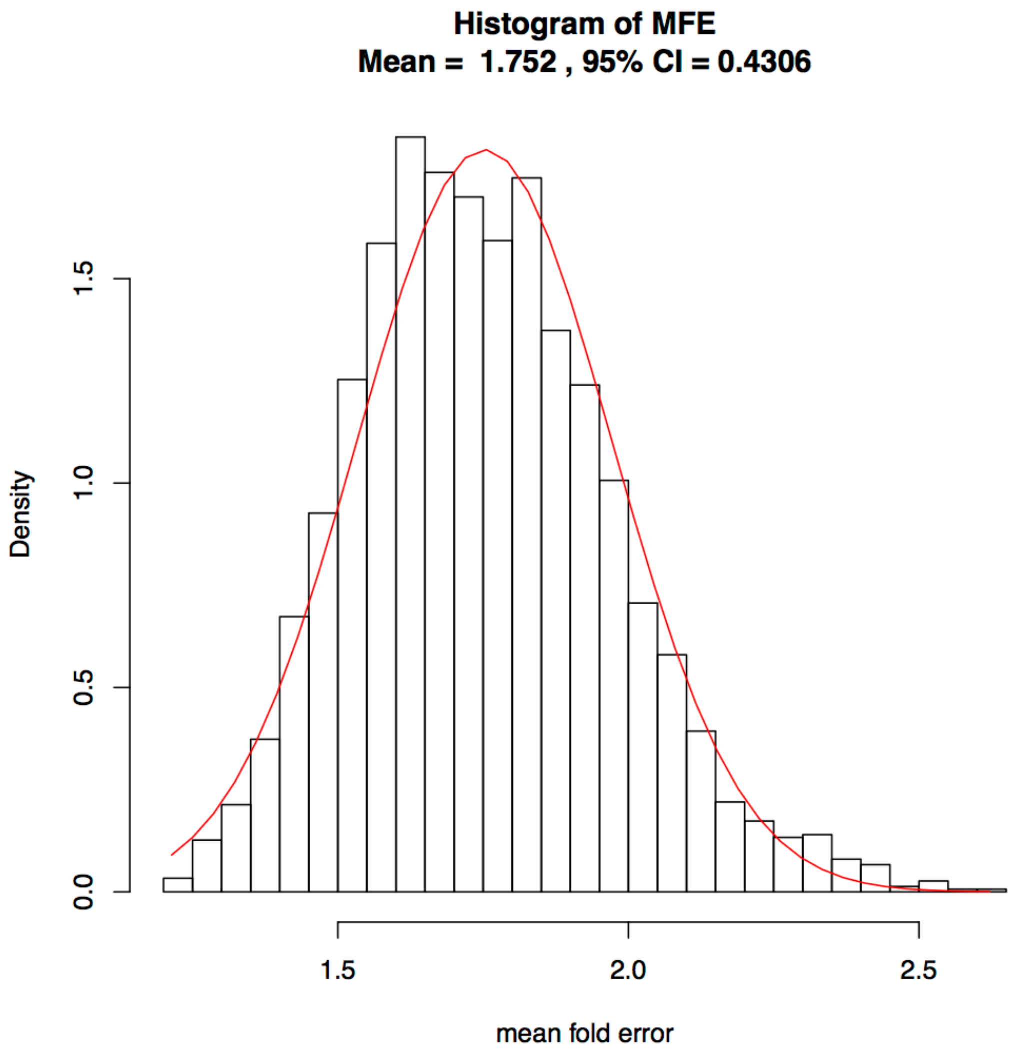

Figure 6: Histogram of the mean fold error for the UPS2_SC dataset. The 95% confidence interval is 0.4306 with a mean of 1.752.

2.3.3 Results for UPS2 MS1 intensity dataset

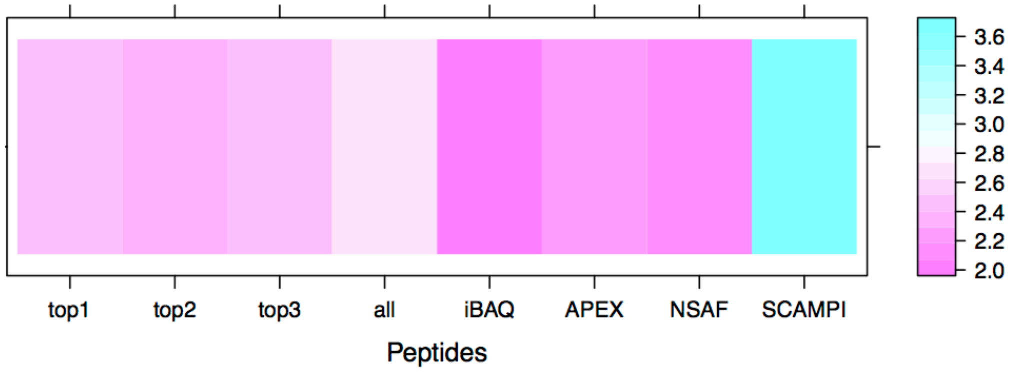

Figure 7: Model selection report for the UPS2_LFQ dataset. The iBAQ protein inference method performed best.

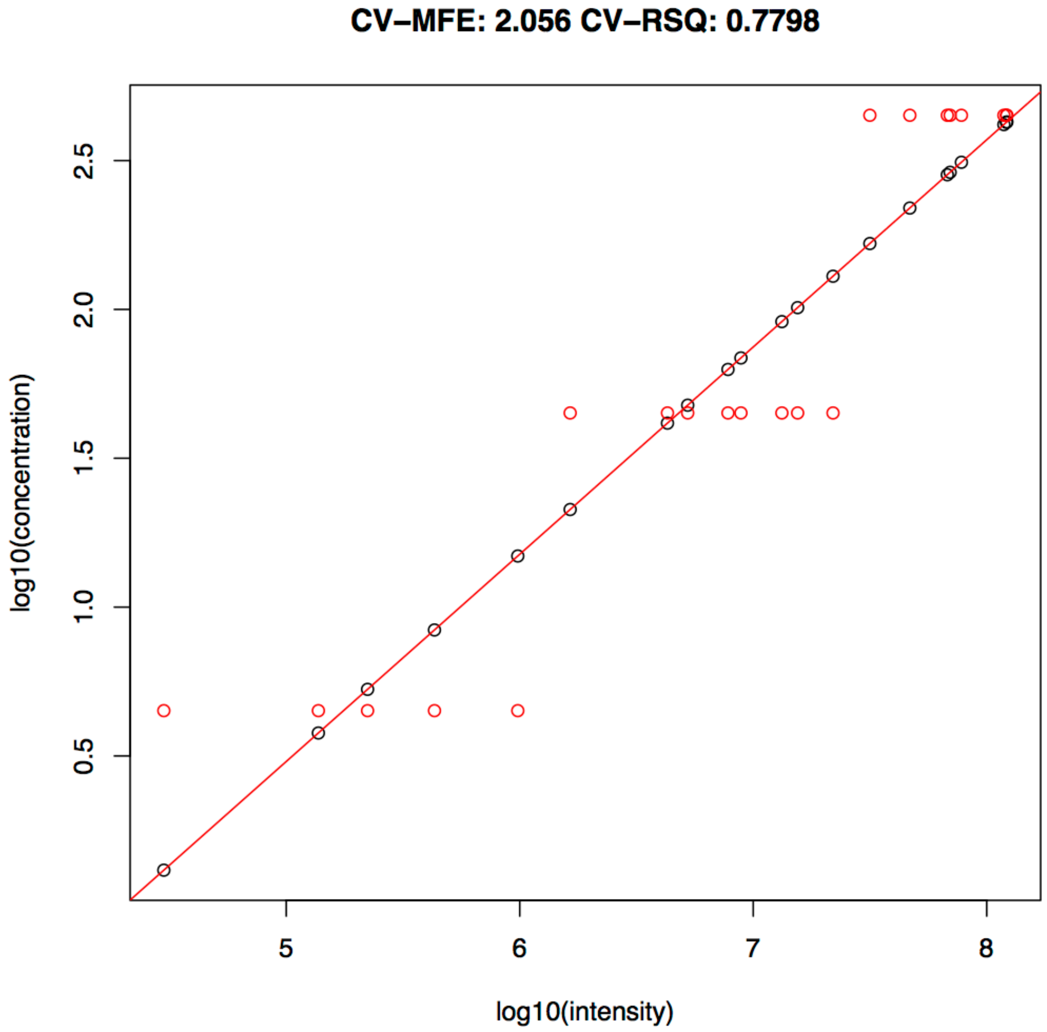

Figure 8: Linear regression plot of log10(intensity) vs log10(concentration) for the UPS2_LFQ dataset. The measured proteins span 2 orders of magnitude with a cross-validated mean-fold error of 2.056.

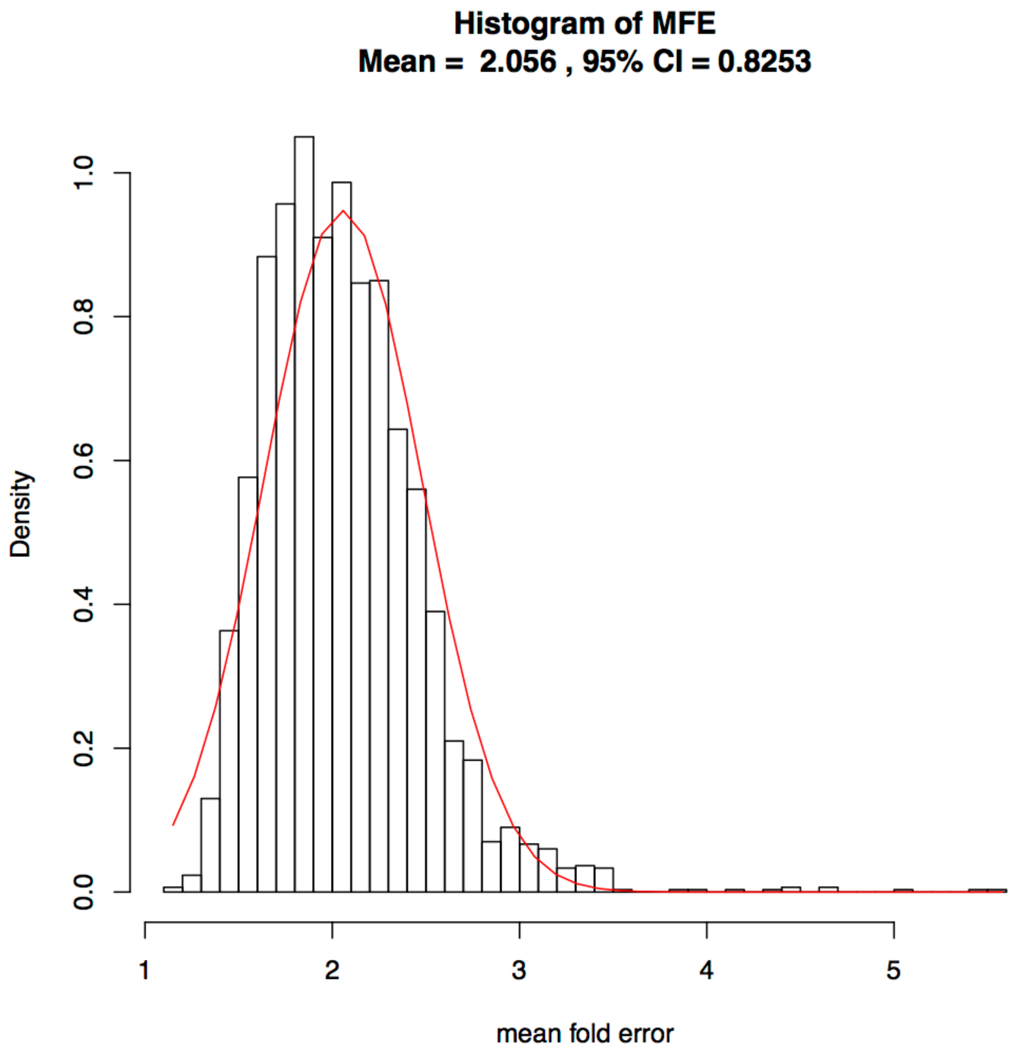

Figure 9: Histogram of the mean fold error for the UPS2_LFQ dataset. The 95% confidence interval is 0.8253 with a mean of 2.056.

3 aLFQ data input formats

3.1 Data import from quantitative MS analysis software

Quantitative results from different MS data analysis software can directly be imported using the import module from aLFQ. Currently, conversion from the output formats of OpenSWATH (Roest *et al.*), OpenMS (Weisser *et al.*, 2013), mProphet (Reiter *et al.*, 2011), Skyline (MacLean *et al.*, 2010) and Abacus (Fermin *et al.*, 2011) is supported directly, omitting any further data formatting or editing step. Table 3 lists the necessary export settings for the supported software packages.

Software	Export
**Abacus**	Default report
**OpenMS**	ProteinQuantifier: “peptides.csv”
**OpenSWATH**	“OpenSWATH_with_dscore.csv”
**mProphet**	“mProphet_bestpeakgroups.xls”
**Skyline**	“Transition Results” report

Table 3: Export settings for primary MS data analysis software packages.

However, also quantitative results from any other software tool can be analyzed using aLFQ, if the data contains all necessary information and has been converted into the generic aLFQ format as described below.

3.2 Targeted MS2-level quantification (SRM)

The data structure for targeted MS2-level datasets represents a table containing the following column header: "run_id" (freetext), "protein_id" (freetext), "peptide_id" (freetext), "transition_id" (freetext), "peptide_sequence" (unmodified, natural amino acid sequence in 1-letter nomenclature), "precursor_charge" (positive integer value), "transition_intensity" (positive non-logarithm floating value) and “concentration” (calibration: positive non-logarithm floating value, prediction: “?”).

3.3 Shotgun MS1-level quantification / Shotgun spectral counts

The data structure for MS1-level intensity / spectral counts datasets represents a table containing the columns "run_id" (freetext), "protein_id" (freetext), "peptide_id" (freetext), "peptide_sequence" (unmodified, natural amino acid sequence in 1-letter nomenclature), "precursor_charge" (positive integer value), "peptide_intensity" (positive non-logarithm floating value) and “concentration” (calibration: positive non-logarithm floating value, prediction: “?”). It should be noted, that the spectral count value is also represented by “peptide_intensity”.

3.4 Experimentally determined anchor protein concentrations

To add experimentally determined anchor protein concentrations, a CSV file must be provided with the columns “run_id” (optional, freetext), “protein_id” (freetext) and “concentration” (positive non-logarithm floating value). Optionally, the concentration of endogenous anchor proteins can automatically be estimated by supplying the spiked-in reference peptides with associated

concentrations. The concentrations of the endogenous peptides are then estimated by the peptide intensity ratios. If multiple peptides per protein are provided, the protein concentration is estimated using the mean of the endogenous peptide concentrations. A CSV file containing the columns "run_id" (optional, freetext), "peptide_id" (freetext) and "concentration" (positive non-logarithm floating value) must be provided.

4 Methods

4.1 Estimation of label-free protein intensities

In bottom-up proteomic approaches, such as shotgun and SRM, not proteins are the measured entity, but peptides. To adapt absolute label-free quantification models from the protein to the peptide level, two assumptions are necessary: First, the theoretical protein intensity can be estimated from the peptide intensities. Second, the theoretical protein response is approximately constant for all proteins in a given proteome.

Different methods for protein intensity estimation are applied within aLFQ:

- TopN: Only the N most intense peptides are considered. The estimator for the protein intensity is the mean of the N measured peptide intensities. (Silva *et al.*, 2006; Malmstrom *et al.*, 2009; Ludwig *et al.*, 2012)
- iBAQ: All peptides are considered. The estimator for the protein intensity is the sum of all measured peptide intensities divided by the number of theoretical fully tryptic peptides between 6 and 30 amino acids for the protein. (Schwanhausser *et al.*, 2011)
- APEX: All peptides are considered. The estimator for the protein intensity is the sum of all spectral counts for the protein multiplied with the probability of detection, normalized by the sum of the predicted probability of observation of all tryptic peptides for the protein. (Lu *et al.*, 2006)
- NSAF: All peptides are considered. The estimator for the protein intensity is the sum of all spectral counts for the protein divided by the number of protein amino acids. (Zybailov *et al.*, 2006)
- SCAMPI: All peptides including those shared between different proteins are considered. The protein intensity is estimated using markovian-type assumptions and parameter estimation. (Gerster *et al.*, 2014)

4.3 Protein concentration estimation using total protein concentration

Label-free protein intensity values can be transferred into absolute protein concentrations by distributing the total protein concentration per cell among all quantified proteins according to their MS intensities. However, this approach requires a correct estimate of the total cellular protein concentration as well as a (as good as) complete proteomic analysis.

$$concentration_{protein} = \frac{intensity_{protein}}{total\_intensity_{protein}} * total\_concentration_{protein}$$

Equation 1: Protein concentration estimation using the total protein concentration.

For the APEX method implemented within aLFQ normalization is carried out by assuming that probabilities from ProteinProphet above the threshold can be rounded to 1.0, because the dataset was filtered using an FDR cutoff instead of probability.

$$concentration_{protein} = \frac{intensity_{protein}}{O_{protein} * \sum_{k=1}^{\#observed\ proteins} \frac{intensity_k}{O_k}} * total\_concentration_{protein}$$

Equation 2: Protein concentration estimation using APEX (Lu *et al.*, 2006). Intensity being the total assigned number of spectra and O being the sum of the machine learning scores of all theoretical peptides of a protein.

4.4 Protein concentration estimation using linear correlation to anchor proteins.

To date, most published absolute label-free protein abundance estimation approaches for mass spectrometry are based on a linear regression between the measured label-free protein intensity and the absolute protein concentration:

$$\log(concentration_{protein}) = \alpha + \beta * \log(intensity_{protein}) + \varepsilon$$

Equation 3: Absolute label-free protein abundance estimation using linear regression. α and β being parameters depending on experimental conditions and ε being the normally distributed error term with mean zero and constant variance.

To calibrate α and β, the concentrations of a few anchor proteins must be known. Accurate measurement of those anchor proteins can be carried out using any absolute quantification technology, however, most frequently SIS peptides are used and spiked into the sample. The concentrations of the corresponding proteins are inferred by the intensity ratio between reference and endogenous peptide. The SIS peptides are selected for proteins of different concentrations to cover a maximal dynamic range.

5 References

Deutsch,E.W. *et al.* (2010) A guided tour of the Trans-Proteomic Pipeline. *PROTEOMICS*, **10**, 1150–1159.

Fermin,D. *et al.* (2011) Abacus: A computational tool for extracting and pre-processing spectral count data for label-free quantitative proteomic analysis. *PROTEOMICS*, **11**, 1340–1345.

Geer,L.Y. *et al.* (2004) Open Mass Spectrometry Search Algorithm. *J. Proteome Res.*, **3**, 958–964.

Gerster,S. *et al.* (2014) Statistical approach to protein quantification. *Molecular \& Cellular Proteomics*, **13**, 666–677.

Keller,A. *et al.* (2005) A uniform proteomics MS/MS analysis platform utilizing open XML file formats. *Molecular Systems Biology*, **1**, 2005.0017.

Keller,A. *et al.* (2002) Empirical statistical model to estimate the accuracy of peptide identifications made by MS/MS and database search. *Anal. Chem.*, **74**, 5383–5392.

Lam,H. *et al.* (2008) Building consensus spectral libraries for peptide identification in proteomics. *Nat Meth*, **5**, 873–875.

Lew,J.M. *et al.* (2011) TubercuList - 10 years after. *Tuberculosis*, **91**, 1–7.

Lu,P. *et al.* (2006) Absolute protein expression profiling estimates the relative contributions of transcriptional and translational regulation. *Nat Biotech*, **25**, 117–124.

Ludwig,C. *et al.* (2012) Estimation of Absolute Protein Quantities of Unlabeled Samples by Selected Reaction Monitoring Mass Spectrometry. *Molecular \& Cellular Proteomics*, **11**, M111.013987–M111.013987.

MacLean,B. *et al.* (2010) Skyline: an open source document editor for creating and analyzing targeted proteomics experiments. *Bioinformatics*, **26**, 966–968.

Malmstrom,J. *et al.* (2009) Proteome-wide cellular protein concentrations of the human pathogen Leptospira interrogans. *Nature*, **460**, 762–765.

Nesvizhskii,A.I. *et al.* (2003) A statistical model for identifying proteins by tandem mass spectrometry. *Anal. Chem.*, **75**, 4646–4658.

Reiter,L. *et al.* (2011) mProphet: automated data processing and statistical validation for large-scale SRM experiments. *Nat Meth*, **8**, 430–435.

Roest,H.L. *et al.* A tool for the automated, targeted analysis of data-independent acquisition (DIA) MS-data: OpenSWATH. *Nat Biotechnol.* in press.

Schwanhausser,B. *et al.* (2011) Global quantification of mammalian gene expression control. *Nature*, **473**, 337–342.

Shteynberg,D. *et al.* (2011) iProphet: multi-level integrative analysis of shotgun proteomic data improves peptide and protein identification rates and error estimates. *Molecular \& Cellular Proteomics*, **10**, M111.007690.

Silva,J.C. *et al.* (2006) Absolute quantification of proteins by LCMSE: a virtue of parallel MS acquisition. *Mol. Cell Proteomics*, **5**, 144–156.

Tabb,D.L. *et al.* (2007) MyriMatch: Highly Accurate Tandem Mass Spectral Peptide Identification by Multivariate Hypergeometric Analysis. *J. Proteome Res.*, **6**, 654–661.

Weisser,H. *et al.* (2013) An automated pipeline for high-throughput label-free

quantitative proteomics. *J. Proteome Res.*, 130208071745007.
Zybailov,B. *et al.* (2006) Statistical Analysis of Membrane Proteome Expression Changes in Saccharomyces c erevisiae. *J. Proteome Res.*, **5**, 2339–2347.
